# Independent monitor unit verification for dynamic flattened beam plans on the Halcyon linac

**DOI:** 10.1002/acm2.13807

**Published:** 2022-10-20

**Authors:** Kazuki Kubo, Mikoto Tamura, Kenji Matsumoto, Masakazu Otsuka, Hajime Monzen

**Affiliations:** ^1^ Department of Medical Physics Graduate School of Medical Sciences Kindai University Osaka‐sayama Osaka Japan; ^2^ Department of Radiology Center Kindai University Hospital Osaka‐sayama Osaka Japan

**Keywords:** dynamic beam flattening, halcyon, in‐air output, monitor unit verification

## Abstract

Independent monitor unit verification (MUV) methods for the dynamic beam‐flattening (DBF) technique have not been established. The purpose of this study was to clarify whether MU values for the DBF technique can be calculated using in‐air and in‐water output ratios (*S*_c_ and *S*_cp_). *S*_c_ and *S*_cp_ were measured in the DBF mode, and the phantom scatter factor (*S*_p_) was calculated. The difference between calculated and planned MUs with square and rectangle fields and clinical plans for different treatment sites was also evaluated. *S*_c_ values for the 4 × 4 to 24 × 24 cm^2^ fields of the distal multi‐leaf collimator (MLC) layer at 2‐cm intervals were 0.887, 0.815, 0.715, 0.716, 0.611, 0.612, 0.511, 0.373, 0.374, 0.375, and 0.374, respectively. No collimator exchange effect was observed. *S*_c_ also depends slightly on the field size of the distal MLC layer. If the distal‐MLC‐layered field size was less than 20% of the corresponding MLC sequence size in the proximal MLC layer, *S*_c_ was affected by >1%, which was compensated using a correction factor (CF). *S*_p_ increased as the field sizes of the MLC sequence and distal MLC leaves increased. MUs calculated using measured *S*_c_, *S*_p_, and CF for square and rectangle fields agreed with planned MUs within ±1.2%. A larger difference (−1.5%) between calculated and planned MUs was observed for clinical plans, whereas differences in MUs were within 2 MU for most fields (56 out of 64 fields). MU calculation for the DBF technique can be performed with *S*_c_, *S*_p_, and CF for independent MUV.

## 1 INTRODUCTION

Independent monitor unit verification (MUV) is an efficient quality control (QC) method for detecting pretreatment errors affecting the treatment planning system dose calculations.^1^ A report of Task Group 114 of the American Association of Physicists in Medicine concluded that MUV remains an important element of the radiation therapy QA/QC program that ensures a safe and accurate patient treatment in modern radiation treatment plan, which uses volumetric imaging, improved computation algorithm, and heterogeneity correction.^1^ MU calculation for photon beams may be performed using dose per monitor unit at the point of interest, in‐air output ratio (*S*_c_), phantom scatter factor (*S*_p_), and tissue phantom ratio (TPR). These factors depend on the field size because of variation in the scattered radiation originating from the collimator head or the phantom, especially *S*_c_ depends on the field size at the virtual source plane that can be seen at the point of interest (i.e., detector's eye view).^1^, ^2^, ^3^, ^4^, ^5^ The dosimetric function values for irregular fields are approximated by using the equivalent field size, which is defined as the square field with the same depth–dose characteristics as the irregular field.^6^ The equivalent field sizes are determined from the blocked field area of the jaws for *S*_c_ and multi‐leaf collimator (MLC) for *S*_p_.^7^, ^8^, ^9^, ^10^, ^11^, ^12^

A new radiotherapy delivery system, the Halcyon (Varian Medical Systems, Palo Alto), was introduced into clinical practice.^13^ Halcyon is designed for faster delivery and higher throughput using a single 6‐MV flattening‐filter‐free (FFF) beam equipped with a dual‐layer MLC and a maximum field size of 28 × 28 cm^2^ compared with a conventional treatment unit.^14^, ^15^ In Halcyon version 2.0, dynamic beam flattening (DBF) is used to mimic flattened beams for conventional radiation therapy treatments.^16^ In this technique, the distal layer of the MLC leaves is used to define a static MLC aperture, and the proximal layer is used to flatten the FFF beam profile with a sliding‐window leaf motion. Halcyon can be used to deliver all types of treatments; however, the independent verification of MU calculations has not been established for DBF plans. Different treatment planning systems cannot be used to verify MU values for DBF plans because only Eclipse (Varian Medical Systems, Palo Alto) supports the creation of plans for the Halcyon. Therefore, DBF plans must be verified with measurements obtained for patient‐specific QC, similar to intensity‐modulated radiation therapy or volumetric modulated arc therapy pretreatment verification.^17^

The purpose of this study was to clarify whether MU for the DBF technique can be calculated with *S*_c_ and *S*_p_ for MUV and to propose a simple MUV method for DBF.

## 2 METHODS

### 2.1 Dynamic beam flattening (DBF)

The Halcyon MLC system features a unique stacked‐and‐staggered dual‐layered design consisting of a distal and a proximal layer. The distal layer, placed further from the source, comprises two banks with 28 leaves each. The proximal layer, placed closer to the source, comprises two banks with 29 leaves each.^18^ The layers are located with a 0.5‐cm offset in the direction perpendicular to the traveling direction of the leaves.^19^ The width of all leaves is 1 cm at the isocenter plane.

**FIGURE 1:**
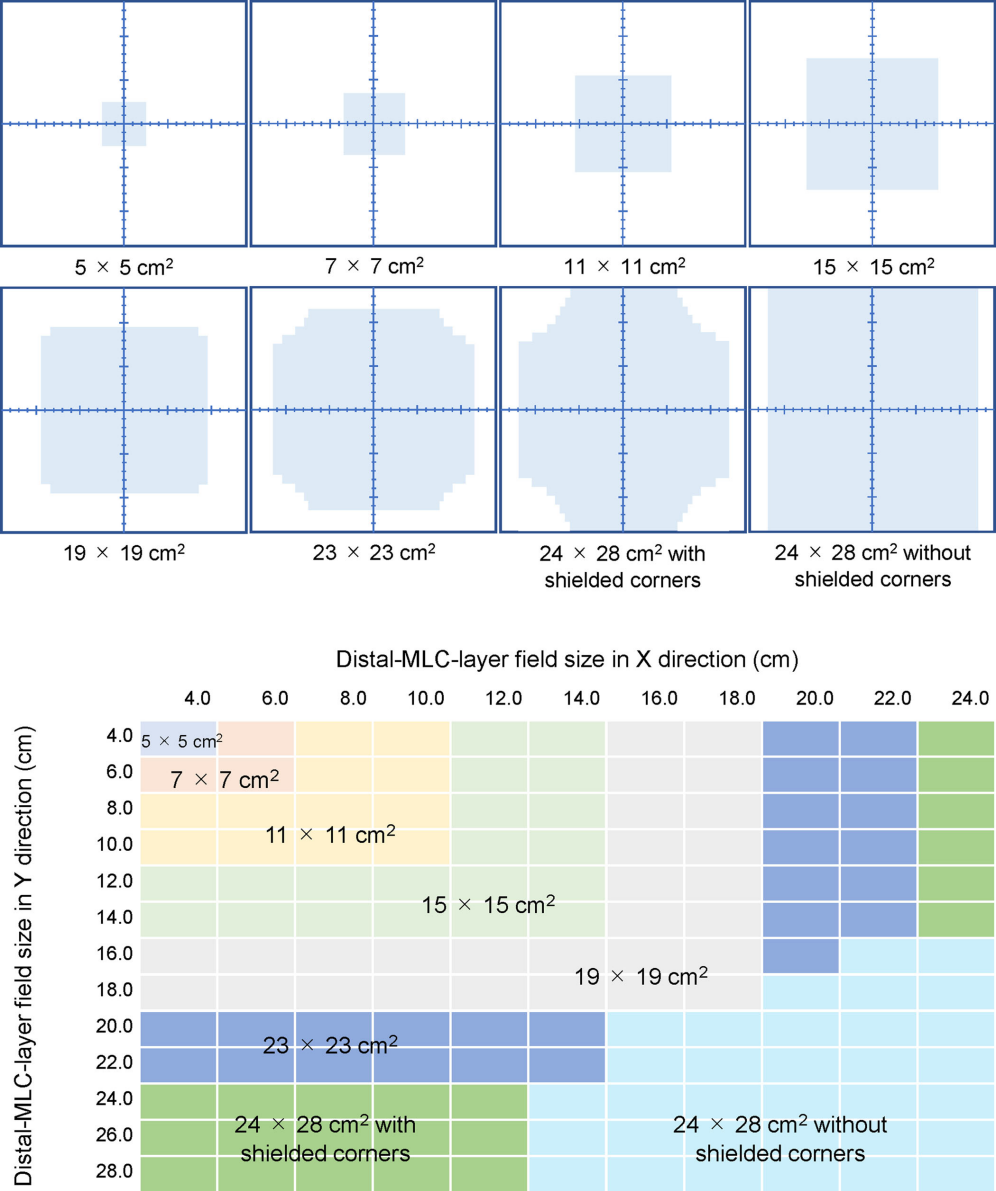
Dynamic beam flattening multi‐leaf collimator (MLC) sequence boundaries in beam's‐eye‐view and relationship between distal‐MLC‐layered field size and MLC sequence selected by the system automatically

In the DBF technique, the proximal layer of the MLC leaves moves according to predefined MLC sequences to compensate the FFF beam profile. The MLC sequence has predefined sweeping pattern with numbers of control points. Predefined MLC sequences are for the following field sizes (*X* × *Y*): 5 × 5, 7 × 7, 11 × 11, 15 × 15, 19 × 19, 23 × 23, and 24 × 28 cm^2^, where *X* and *Y* are the directions parallel and perpendicular to the traveling direction of the leaves, respectively (Figure 1). Those are only available predefined ones from the system. Two MLC sequences for the 24 × 28 cm^2^ field are provided: with and without shielded corners, which are used to provide better protection against leakage at the closing positions of the leaf pairs in the distal MLC layer. When user shapes a field by the distal MLC layer, the MLC sequence for the proximal layer is automatically selected by the system. The system selects the smallest possible MLC sequence for the aperture defined by the user. DBF aims for a flat profile at a depth of 10 cm, where flatness is defined as a deviation of no more than ±3% from the dose at the central axis for 80% of the central field of the 10 × 10 to 22 × 22 cm^2^ fields.^13^, ^20^ The flatness for the field sizes smaller than 10 × 10 cm^2^ is not defined in manufacturer's reference guide.^20^

### 2.2 Measurement of in‐air output ratio (*S*_c_)

In‐air outputs for the distal‐MLC‐layered field sizes of 4 × 4 to 24 × 24 cm^2^ at 2‐cm intervals were measured at isocenter using an 0.6 cm^3^ ionization chamber (PTW, Freiburg) inserted into a cylindrical mini‐phantom (Qualita, Nagano) with 4 cm in diameter and 20 cm in length. The ion chamber was placed 10 cm below the surface of the mini‐phantom. As MLC sequences of the 23 × 23 and 24 × 28 cm^2^ fields with shielded corners are not used for a square field (Figure 1), in‐air outputs for the 20 × 16 and 12 × 24 cm^2^ fields of the distal MLC layer were measured instead of them. To calculate *S*_c_, all in‐air outputs were normalized to the in‐air output of the 10 × 10 cm^2^ static field produced in the FFF mode. All measurements were repeated three times with 100 MU, and the values were averaged.

In addition, in‐air outputs for the 14 × 4 , 4 × 14 , 18 × 4, and 18 × 4 cm^2^ fields were measured to determine the magnitude of the collimator exchange effect.^21^

In‐air outputs for rectangle fields were measured to determine the effect of the field size of the distal MLC layer on *S*_c_. The *X* side of the field was fixed at 4 cm for all rectangle fields, and the *Y* side of the field was changed from 8 to 28 cm at 2‐cm intervals. Because the diameter of the mini‐phantom used for this study was 4 cm, lateral equilibrium was not achieved for smaller fields (<4 × 4 cm^2^).

### 2.3 Measurement of in‐water output ratio (*S*_cp_) and calculation of a phantom scatter factor (*S*_p_)

In‐water output measurements were carried out using the same 0.6‐cm^3^ ionization chamber placed at a depth of 10 cm in a 35 × 35 × 40 cm^3^ water phantom (Qualita, Nagano) with a source‐to‐surface distance (SSD) of 90 cm for the same field sizes as the *S*_c_ measurements. This measurement depth was determined to meet the calibration depth for the Halcyon in our center. To calculate *S*_cp_, all in‐water outputs were normalized to the in‐water output of the 10 × 10 cm^2^ static field produced in the FFF mode. Thereafter, *S*_p_ was calculated using *S*_cp_ and *S*_c_ for each field size according to the following equation:

(1) $$\;S_{\mathrm{cp}}=S_{c}\;\times S_{p}\cdots$$

### 2.4 Comparison of calculated and planned monitor units (MUs)

*S*_c_ and *S*_p_ obtained in this study were evaluated by comparing the calculated and planned MUs. The planned MUs were obtained from Eclipse treatment planning system version 15.6 (Varian Medical Systems, Palo Alto). The MUs for square and rectangle fields were calculated to deliver 100 cGy to the isocenter at a depth of 10 cm with an SSD of 90 cm in a water‐cube phantom. The dose calculation algorithm, Anisotropic Analytical Algorithm (AAA) version 15.6.06 (Varian Medical Systems, Palo Alto), was used without heterogeneity correction. For MUV, the determination of the field size for *S*_p_ was based on the field size of the distal MLC leaves. The method of Sterling et al.^22^ was used to calculate the equivalent square‐field size. Determination of *S*_c_ was based on the DBF MLC sequence used for the field. The equation for MU is as follows:

(2) $$\begin{array}{c} \mathrm{MU}=\frac{D}{D_{0}\;\cdot \;S_{c}\;\cdot \;S_{p}\left(r_{d}\right)\;\cdot \;\mathrm{TPR}\left(d,r_{d}\right)\;\cdot \;\mathrm{CF}\;\cdot \;{\left(\frac{{\mathrm{SSD}}_{0}+d_{0}}{\mathrm{SAD}}\right)}^{2}}\;\cdots \;\;\; \end{array}$$

where *D*_0_ is the dose per MU at the normalization depth (10 cm) for 10 × 10 cm^2^, *D* is the absorbed dose at the point of interest, and *r*_d_ is the equivalent square of the treatment aperture defined by collimating devices projected to the plane normal to the central axis containing the point of calculation at a depth *d*. In this study, the point of interest and calculation depth were 10 cm. The TPR was 1.0, and the effect of TPR errors on the accuracy of *S*_c_ and *S*_p_ was minimized. CF is a correction factor for *S*_c_, introduced to consider the effect of the field size of the distal MLC layer on *S*_c_. The details of CF are described in the following subsection. The standard SSD (SSD_0_) is the distance along the central axis from the physical source to the patient/phantom surface under normalization conditions, and *d*_0_ is the normalization depth. The source‐to‐axis distance (SAD) is the distance between the X‐ray physical source position and the isocenter. The values of SSD_0_ + *d*_0_ and SAD were equal in this study.

### 2.5 Correction factor for in‐air output on the Halcyon linac

The machine output can deviate from the predicted output when the area of the field shaped by the MLC is less than ∼50% of the original field area.^23^ Nara et al. introduced a new method to correct *S*_c_ for fields shaped by MLC in which the CF is determined by the ratio of *S*_c_ for an MLC square field to *S*_c_ for a jaw square field.^24^ The Halcyon does not have a jaw, but larger differences between the proximally and distally defined field areas may provide different *S*_c_. We also attempted to correct *S*_c_ for rectangle fields in the Halcyon by referring to the method of Nara et al. The ratio of *S*_c_ for a rectangle field to *S*_c_ for a square field was calculated using the results of *S*_c_ measurements. All field area were defined at the isocenter distance. These ratios were plotted against the ratio of the field area of the MLC sequence defined by the proximal leaves to the field area of the distal MLC leaves, and a regression curve was determined for this plot. The field area of the MLC sequence was defined based on the sequence boundaries shown in Figure 1. Thus, an *S*_c_ CF can be applied according to the ratio of the field area of the MLC sequence to the field area of the distal MLC leaves.

### 2.6 Comparison of calculated and planned monitor units (MUs) with clinical plans

For further comparison, the difference between calculated and planned MUs in irregular‐shaped fields was evaluated. A total of 64 fields was collected from 19 clinical plans for patients with metastatic brain tumor (2 plans), esophagus cancer (2 plans), lung cancer (3 plans), breast cancer (3 plans), pancreas and liver cancer (3 plans), pelvic cancer (4 plans), and metastatic spinal tumor (2 plans). All plans were created with two to five fields, and AAA was used to calculate the dose distribution. Prescription doses were 2–3 Gy at the isocenter. To eliminate the error caused by patient geometry, all fields were recalculated with a water‐cube phantom. The calculation point was placed at a depth of 10 cm. MUV was performed using the same geometry without homogeneity correction. The method of Onai et al.^25^ was used to calculate the equivalent square‐field size for the determination of *S*_p_. Their method is simpler than Clarkson's method^26^ to estimate *S*_p_ for irregular‐shaped fields at the isocenter.

### 2.7 Difference between MUV and portal dosimetry results for clinical plans

Our MUV was performed for some plans of the spine and whole brain, which were evaluated using portal dosimetry (PD; Varian Medical Systems, Palo Alto),^27^, ^28^ and then delivered to patients in the clinic. The calculated and measured fluences were compared in the PD workspace incorporated in Eclipse. The results of the gamma evaluation with a dose difference of 3%, a distance‐to‐agreement of 2 mm,^29^ and a low‐dose threshold of 10% were compared with the results of MUV. The MUs for fields were calculated using patient geometry and depths without inhomogeneity correction, and the conditions were more advanced than those of this study.

## 3 RESULTS

### 3.1 Measurement of in‐air output ratio (*S*_c_)

**FIGURE 2:**
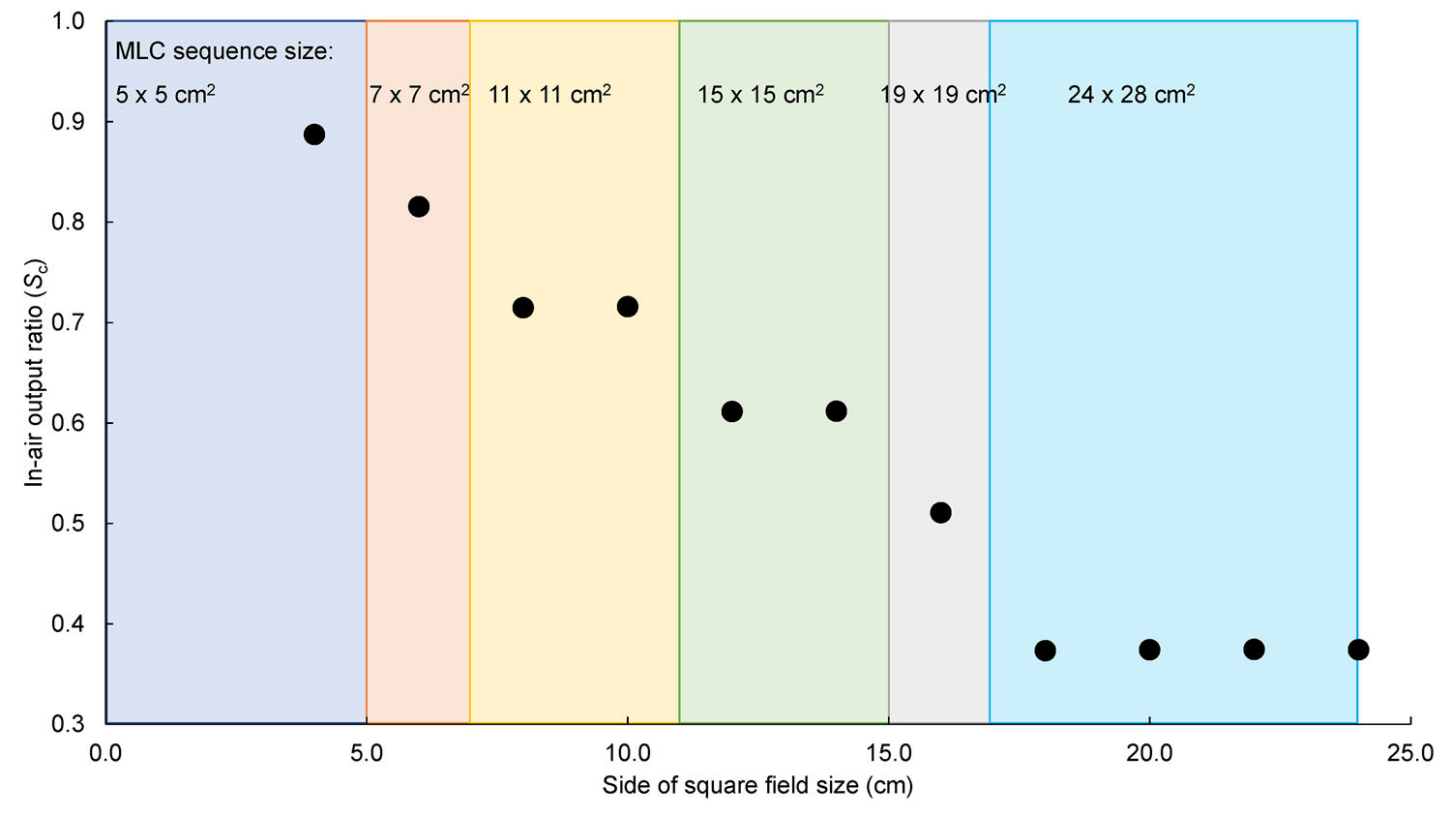
Ratio of in‐air output (*S*_c_) for square fields with size of multi‐leaf collimator (MLC) sequence used. MLC sequences for the 23 × 23 and 24 × 28 cm^2^ fields with shielded corners were not used for the square fields. To obtain *S*_c_ in these MLC sequence fields, 20 × 16 and 12 × 24 cm^2^ fields were used, and the values of *S*_c_ were 0.411 and 0.433, respectively. Using the same MLC sequence (shown in this figure as different colors), the same value of *S*_c_ was observed even if the field size of the distal MLC leaves was different.

*S*_c_ values were 0.887, 0.815, 0.715, 0.716, 0.611, 0.612, 0.511, 0.373, 0.374, 0.375, and 0.374 for the 4 × 4 to 24 × 24 cm^2^ fields of the distal MLC layer at 2‐cm intervals, respectively (Figure 2). *S*_c_ values were 0.411 and 0.434 for the 20 × 16 and 12 × 24 cm^2^ fields of the distal MLC leaves, respectively.

*S*_c_ values for the 14 × 4 and 4 × 14 cm^2^ fields were 0.605 and 0.606, respectively. *S*_c_ values for the 18 × 4 and 4 × 18 cm^2^ fields were 0.503 and 0.504, respectively. The collimator exchange effect was very insignificant.

**FIGURE 3:**
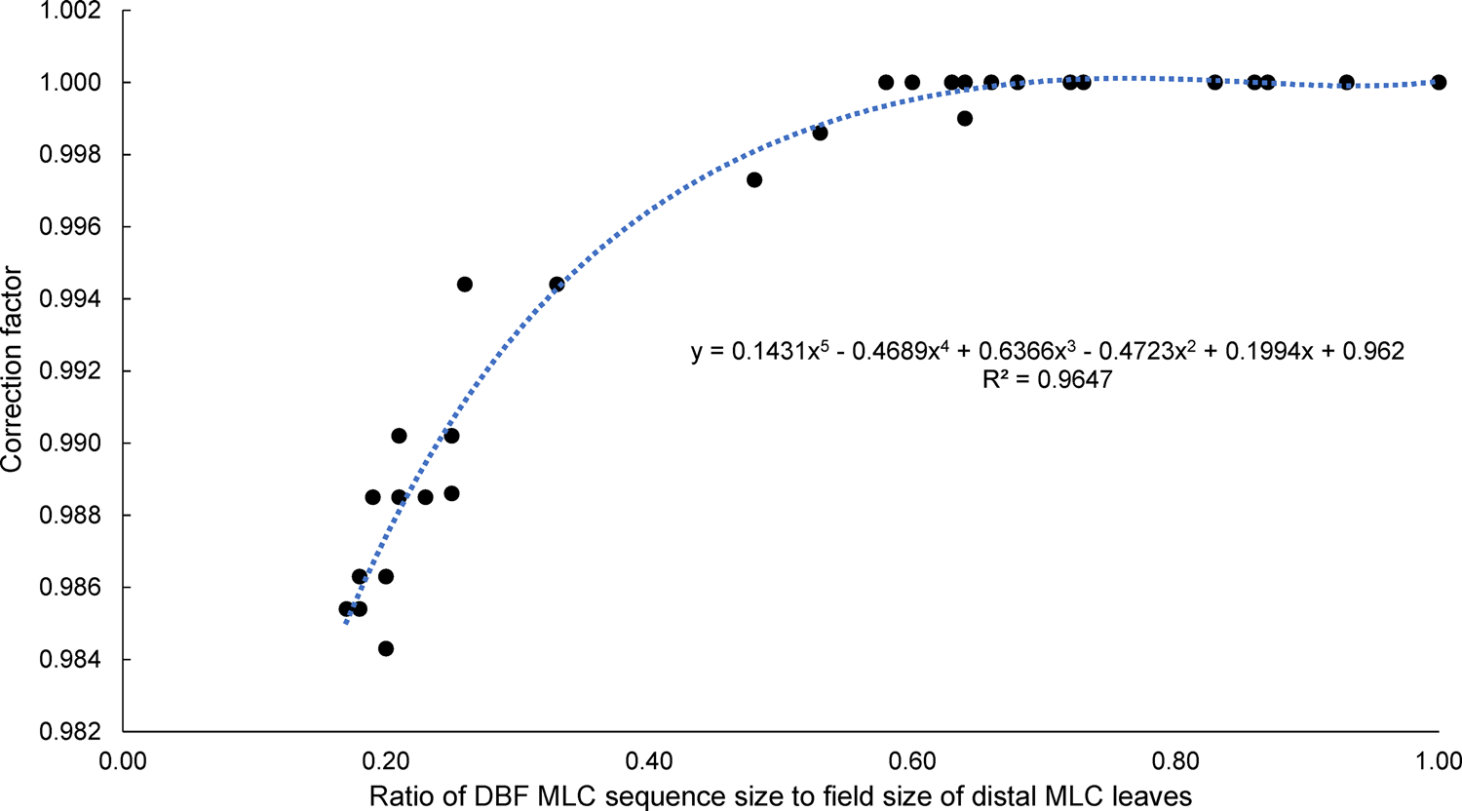
Correction factor of *S*_c_ as a function of the ratio of multi‐leaf collimator (MLC) sequence size to field size of distal MLC leaves. There were 30 cases in this figure, and all values of measured field sizes in this study were plotted: 11 square fields, 19 rectangular fields.

To investigate the effect of the field size of the distal MLC layer on *S*_c_, measurements were conducted with one side of each field (*X* side) set to 4 cm. Figure 3 shows the ratio of *S*_c_ for a rectangle field to *S*_c_ for a square field. There were 30 cases in Figure 3, and all values of measured field sizes in this study were plotted. *S*_c_ changed by more than −1% when the ratio of the rectangle‐field area to square‐field area was less than 20%. A polynomial regression curve was added to the plot in Figure 3, and a CF for *S*_c_ depending on the ratio of the proximal and distal field areas was determined.

### 3.2 Measurement of in‐water output ratio (*S*_cp_) and calculation of a phantom scatter factor (*S*_p_)

**FIGURE 4:**
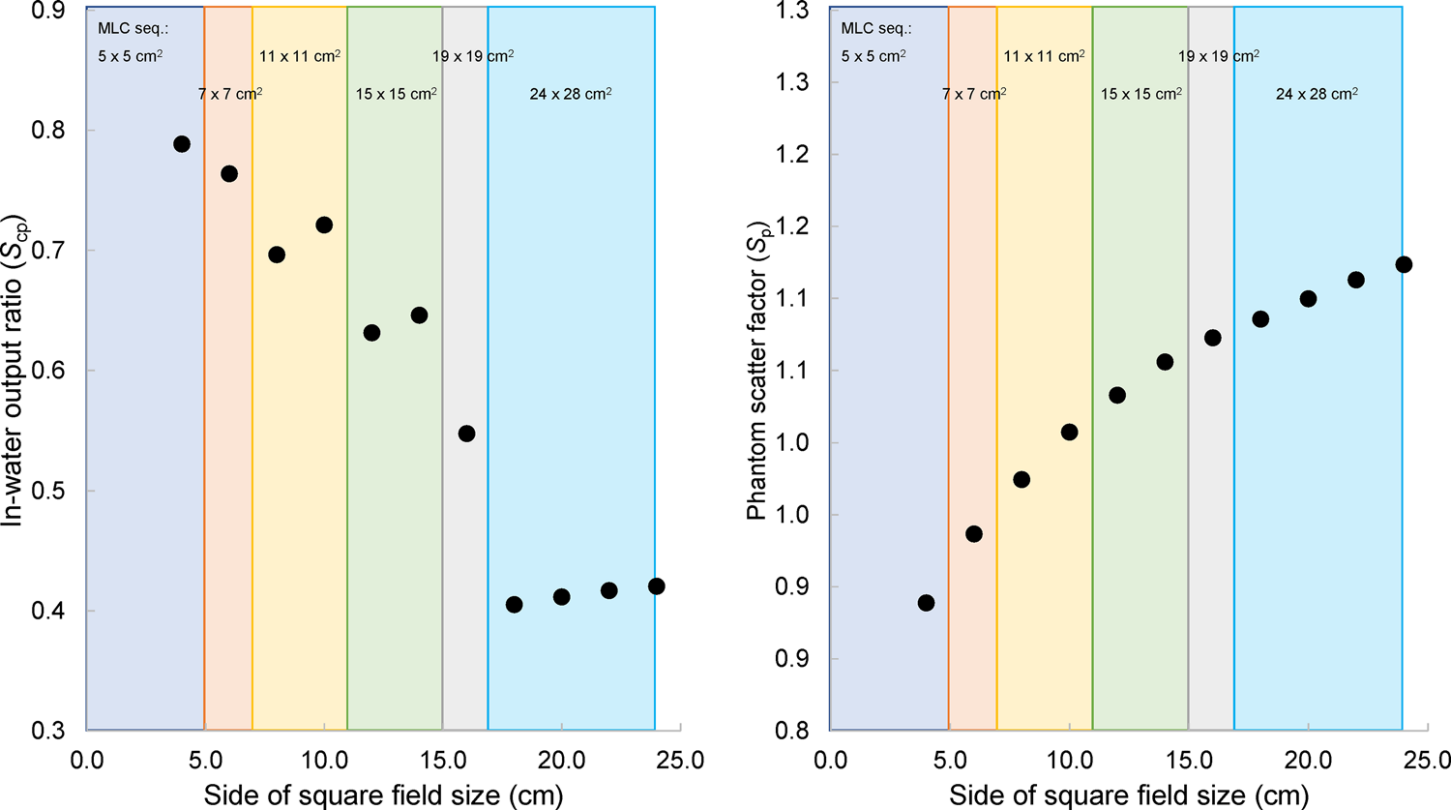
(a) Ratio of in‐water output (*S*_cp_) for square fields with size of multi‐leaf collimator (MLC) sequence used and (b) phantom scatter factor (*S*_p_) for square fields with size of MLC sequence used

Parts (a) and (b) of Figure 4 show the results for *S*_cp_ and *S*_p_, respectively. *S*_cp_ decreased as the field size of the MLC sequence increased. *S*_cp_ slightly increased as the field size of the distal MLC leaves increased when the same MLC sequence was used for beam flattening. *S*_p_ increased as the field sizes of the MLC sequence and distal MLC leaves increased.

### 3.3 Comparison of calculated and planned monitor units (MUs)

Calculated and planned MUs for square and rectangle fields, summarized in Table 1, were compared. The largest difference (−1.2%) between the calculated and planned MUs was observed for the 6 × 28 and 8 × 28 cm^2^ fields. Calculated and planned MUs for most field sizes agreed (within ±1.0%).

**TABLE 1:** Difference (%) of calculated MUs relative to planned MUs from Eclipse version 15.6 for square and rectangle fields at a depth of 10 cm with a source‐to‐surface distance of 90 cm

	Field size of *X* direction (cm)										
Field size of *Y* direction (cm)	4.0	6.0	8.0	10.0	12.0	14.0	16.0	18.0	20.0	22.0	24.0
4.0	0.0	−0.4	0.1	−0.1	−0.1	−0.3	−0.3	−0.5	−0.6	−0.7	−0.6
6.0	−0.3	0.0	0.1	0.0	−0.2	−0.3	−0.5	−0.7	−0.8	−1.0	−1.0
8.0	0.2	0.1	0.0	0.2	−0.2	−0.3	−0.5	−0.6	−0.8	−0.9	−0.9
10.0	0.0	0.0	0.2	0.5	0.0	0.0	−0.4	−0.4	−0.6	−0.7	−0.7
12.0	−0.0	−0.2	−0.2	0.0	0.1	0.2	−0.2	−0.1	−0.4	−0.4	−0.4
14.0	−0.4	−0.2	−0.3	−0.3	0.0	0.5	0.0	0.1	−0.1	−0.1	−0.1
16.0	−0.2	−0.5	−0.5	−0.4	−0.2	0.0	0.2	0.4	0.1	0.0	−0.1
18.0	−0.4	−0.6	−0.6	−0.5	−0.2	0.1	0.4	−0.2	0.1	0.1	0.1
20.0	−0.5	−0.8	−0.8	−0.7	−0.4	−0.2	0.0	0.1	0.1	0.2	0.2
22.0	−0.7	−1.0	−1.0	−0.8	−0.5	−0.2	−0.1	0.1	0.2	0.2	0.2
24.0	−0.6	−0.9	−0.9	−0.8	−0.5	−0.3	−0.1	0.0	0.2	0.2	0.1
26.0	−0.7	−1.1	−1.1	−0.9	−0.5	−0.4	−0.2	0.0	0.1	0.1	0.0
28.0	−0.8	−1.2	−1.2	−1.0	−0.6	−0.5	−0.2	0.0	0.0	0.0	−0.3

**FIGURE 5:**
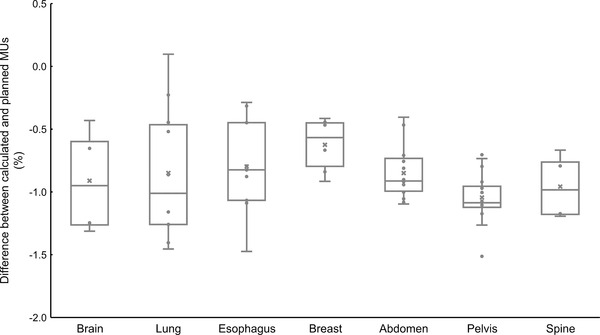
Difference (%) of calculated MUs relative to planned MUs from Eclipse version 15.6 for different treatment sites in clinical plans

Figure 5 shows the results of comparing the calculated and planned MUs for irregular‐shaped fields by case. The largest difference (−1.5%) between calculated and planned MUs was observed for pelvis case, and the smallest difference (0.1%) was observed for lung case. Differences in MUs were within 2 MU for most fields (56 out of 64 fields). The largest difference between calculated and planned MUs was 3.4 MU for brain case. Calculated MUs were systematically lower than planned MUs for most fields.

### 3.4 Difference between MUV and portal dosimetry results for clinical plans

The results of MUV were 1.46% and 0.35% for two fields in the spine plan and 1.51% and −1.05% for two fields in the whole brain plan compared with planned MUs for the fields, whereas the gamma pass rates were >99% for all fields.

## 4 DISCUSSION

The results of this study demonstrate that *S*_c_ can be determined from the DBF MLC sequence, and *S*_p_ can be determined from the field size of the distal MLC leaves in the DBF mode. The MU for a DBF field can be calculated to planned MUs within ±2% using the look‐up tables as independent MUV for conventional linear accelerators.

*S*_c_ was dominated by MLC sequence field size in Figure 1. In a conventional treatment machine with traditional upper and lower jaws, *S*_c_ is determined on the basis of the field size of the collimator jaws. However, if the field size is smaller (<0.5, i.e., smaller than half of the field area) with an MLC than with collimator jaws, the value of *S*_c_ may change.^23^ In the Halcyon, the proximal MLC layer plays a role like the collimator jaws to determine *S*_c_ value for the field size. To calculate MUs, *S*_c_ was selected on the basis of the MLC sequence used for the field, and CF was applied to account for the effect of the field size of the distal MLC leaves. The field size of the distal MLC leaves affected *S*_c_, which depended on the ratio of field area of the DBF MLC sequence to the field area of the distal MLC leaves. Nara et al. attempted to calculate accurate *S*_c_ values using CFs for irregular‐shaped fields with MLC. Therefore, we applied CF to correct *S*_c_ and eliminate the effect of the field size of the distal MLC layer. *S*_p_ increased as the field sizes of the MLC sequence and distal MLC leaves increased. For rectangular fields, the equivalent square field converted from the field size of the distal MLC leaves can be used successfully, even if the proximal MLC leaves move according to the preconfigured MLC sequence during irradiation in the DBF mode. With square and rectangle fields, the difference between calculated and planned MUs was less than 1.5% in this study. With irregular‐shaped fields in clinical plans, the difference between calculated and planned MUs was less than 2%, which was slightly larger than those of square and rectangle fields, and calculated MUs were systematically lower than planned MUs. These discrepancies likely originated in the accuracy of the calculated equivalent square field for the determination of *S*_p_. Moreover, Kim et al. found that measured dosimetric leaf gap sizes for each layer were different compared with defined value in the TPS.^19^ The suboptimal leaf end modeling may affect the results systematically. However, the differences were small. Some previous studies present guidelines for action levels for disagreement between planned and calculated MUs, depending on treatment site, treatment technique, patient geometry, and whether corrections for tissue heterogeneities are used.^1^, ^30^, ^31^ They concluded action levels of 3%–5% were clinically realistic based on collected data from multi‐institutions or the collective experience and expectations of the task group members. The results of this study indicate that our method is sufficiently accurate for MUV of DBF fields in clinical practice. Verifying MU without measurements for patient plans leads to improved workflow in the clinic, thus reducing the waiting time for radiotherapy. Plans for the Halcyon can only be created using Eclipse, and third‐party treatment planning systems cannot be used for MUV. Additionally, all Halcyon treatment plans use dynamic MLC sequences even if the plan is used for conventional radiotherapy. As things stand, the dose in all plans should be measured for patient‐specific QC. Our results support the establishment of independent MUV methods for DBF fields on the Halcyon to detect pretreatment errors affecting the TPS dose calculations.

In this study, the collimator exchange effect was very small for the dual‐layered MLC in the Halcyon. For linear accelerators equipped with conventional jaws, because the upper collimator jaws are closer to the source than the lower jaws, the same opening size made by the upper and lower jaws in the beam's eye view are different when viewed from the perspective of calculations, and thus, the collimator backscattered radiation, which contributes to the monitor unit signal, from each jaw is different. In the Halcyon, the same MLC sequence is used in the proximal MLC layer even if the field size of the distal MLC layer changes, and thus the collimator backscattered radiation from each layer is the same. In addition, FFF beam related to the reduction of head scatter would be also the reason.^32^

In addition, Our MUV method may be applied instead of PD verification for DBF plan in the Halcyon and may lead to the independency of the verification process because the PD is a system provided by the manufacturer.

There are some limitations in this study. Calculation point was the isocenter for all fields, and homogeneity correction was not used. Difference between calculated and planned MUs might indicate a different tendency when a calculation point at off‐axis is used or heterogeneity tissues is considered. To calculate MUs in clinical practice, TPR is required for different calculation depth, but it was out of scope in this study. We further investigate them in the future study. In order to validate whether our method is sufficiently accurate for MUV of DBF fields in clinical practice, the following three steps would be required: (1) factor model design and commissioning using measurements in regular fields, (2) characterization of the differences in MU calculations between the factor model and the TPS, and (3) defining criteria for a clinical MUV procedure based on this characterization/analysis. In this study, we focused on steps (2) and (3), but step (1) was not confirmed with adequately validation set. The beam data to be used for dose calculation for the Halcyon are provided from manufacturer (i.e., representative beam data), and users cannot do any modifications. We assumed that the beam data and measurement in regular fields meet within clinical criteria.

## 5 CONCLUSION

MU calculation for the DBF technique can be performed with *S*_c_, *S*_p_, and CF for independent MUV. This simple method can be used to verify DBF plans, which may reduce the frequency of measurement for patient‐specific QC.

## AUTHOR CONTRIBUTIONS

Kazuki Kubo and Hajime Monzen conceived the idea of the study. Kazuki Kubo, Mikoto Tamura, Kenji Matsumoto, and Masakazu Otsuka significantly contributed to data analysis and interpretation. All authors reviewed the manuscript draft and revised it critically on intellectual content. All authors approved the final version of the manuscript to be published.

## CONFLICTS OF INTEREST

The authors report no conflicts of interest. The authors are responsible for the content and writing of the paper.
